# Association of Branded Prescription Drug Rebate Size and Patient Out-of-Pocket Costs in a Nationally Representative Sample, 2007-2018

**DOI:** 10.1001/jamanetworkopen.2021.13393

**Published:** 2021-06-14

**Authors:** Kai Yeung, Stacie B. Dusetzina, Anirban Basu

**Affiliations:** 1 Kaiser Permanente Washington Health Research Institute, Seattle, Washington; 2 Comparative Health Outcomes, Policy, and Economics Institute, University of Washington, Seattle, Washington; 3 Department of Health Policy, Vanderbilt University School of Medicine, Nashville, Tennessee; 4 Vanderbilt-Ingram Cancer Center, Nashville, Tennessee; 5 National Bureau of Economic Research, Cambridge, Massachusetts

## Abstract

**Importance:**

Over the past decade, branded prescription drug manufacturers have substantially increased list prices while offering larger rebate payments to health care insurers. Whereas larger rebates can partially offset increases in list prices for insurers, patient out-of-pocket costs may be directly associated with list prices for individuals without insurance and indirectly associated with list prices for individuals with insurance through deductibles or coinsurance.

**Objective:**

To investigate the association between rebates and patient out-of-pocket costs and whether this association differs by coverage type (ie, Medicare, commercial, or uninsured) and before and after 2014.

**Design, Setting, and Participants:**

This cross-sectional study was conducted using data from the Medical Expenditure Panel Survey (MEPS) combined with pricing data for single-source branded drugs from SSR Health from 2007 through 2018. The study was conducted among a nationally representative sample of the noninstitutionalized civilian US population. Included individuals were respondents to MEPS with at least 1 prescription for a single-source branded drug who were covered by Medicare or commercial insurance or were uninsured during an entire year. Data analyses were conducted from August 2019 through March 2021.

**Exposures:**

Estimated rebate size.

**Main Outcomes and Measures:**

Out-of-pocket costs per prescription were calculated, adjusting for year and drug.

**Results:**

Among 38 131 individuals with at least 1 prescription, the mean age was 54 years (95% CI, 54 to 55 years), with 22 044 women (57.8%) and 29 086 White individuals (76.3%). The sample included 444 unique drugs with a survey-weighted total of 4.7 billion prescriptions. Estimated mean (SE) rebates increased from $34 ($1) per prescription in 2007 to $374 ($9) per prescription in 2018. The rebate sizes were associated with statistically significant mean out-of-pocket increases per branded prescription of $4 (95% CI, $4 to $4) from 2007 to 2013 and $11 (95% CI, $10 to $12) from 2014 to 2018. From 2014 to 2018, rebate sizes were associated with statistically significant mean increases in out-of-pocket costs per prescription of $13 (95% CI, $12 to $13) for individuals with Medicare, $6 (95% CI, $6 to $7) for individuals with commercial insurance, and $39 (95% CI, $34 to $44) for individuals without insurance. After adjusting for list prices, there was no association between rebates and out-of-pocket costs, with a change from 2014 to 2018 of −$0.01 (95% CI, −$0.04 to $0.02).

**Conclusions and Relevance:**

These findings suggest that drug manufacturers may have provided larger rebates to insurers primarily by increasing list prices and that individuals without insurance had greater cost increases. The results emphasize the need for policy solutions that decouple list prices and out-of-pocket costs.

## Key Points

**Question:**

Are prescription drug rebates associated with increased patient out-of-pocket costs?

**Findings:**

In this cross-sectional study of estimated rebates for 444 unique branded drugs with prescriptions filled by 38 131 unique individuals, increased rebate sizes were associated with increased out-of-pocket costs for those with Medicare, commercial insurance, or no insurance. Associations between rebates and out-of-pocket costs were associated with simultaneous increases in list prices.

**Meaning:**

These findings suggest that while drug manufacturers may increase list prices in order to offer larger rebates to insurers, such increases were associated with increased out-of-pocket costs, especially among individuals without insurance.

## Teaser

This cross-sectional study investigates the association of branded prescription drug rebate size and patient out-of-pocket costs.

## Introduction

Controlling prescription drug spending is a national priority for the public,^1^ health care insurers,^2^ and governments.^3^ One way that insurers attempt to slow drug spending growth is by contracting with pharmacy benefit managers (PBMs), who negotiate drug prices on behalf of multiple insurers. These companies negotiate discounts on list prices from drug manufacturers in the form of rebates in exchange for providing favorable insurance coverage. Such rebates can contribute substantially to insurer savings and, therefore, future enrollee premium savings. For instance, Medicare Part D collected $24 billion in rebates in 2018.^4^ However, PBMs can keep a portion of the rebates, so stakeholders, including the US Department of Health and Human Services, have raised concerns that manufacturers may increase list prices to offer larger rebates to PBMs.^5,6^ Indeed, list prices for branded drugs increased by 159% from 2007 to 2018.^7^

**Figure 1.**
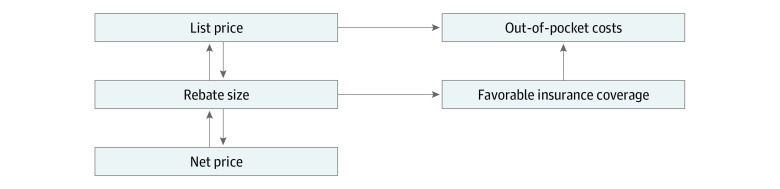
Conceptual Model for Association of Rebates With Out-of-Pocket Costs Through List Prices and Insurance Coverage Generosity Net prices represent the revenue received per unit of drug by a manufacturer that reflects all price reductions, including rebates.

Larger rebates may be associated with out-of-pocket costs via 2 opposing mechanisms (Figure 1). Through increasing list prices, rebates may be positively associated with out-of-pocket costs for individuals without insurance as well as for those with insurance because cost-sharing structures, such as deductibles and coinsurance, require individuals with insurance to pay a portion of list prices. The exposure to list prices for individuals with insurance may be increasing with the increasing use of these cost-sharing structures in Medicare Part D^8,9^ and commercial plans.^10,11^ Alternatively, rebates may be negatively associated with out-of-pocket costs if PBMs provide more favorable insurance coverage (ie, lower cost-sharing charges) in exchange for larger rebates from manufacturers.

Measuring the direction and magnitude of association between rebates and out-of-pocket costs is important because increased out-of-pocket costs are associated with negative outcomes in patient financial burden,^12,13^ medication adherence,^14^ and health in some populations.^15,16,17^ Our primary objective was to investigate the association between rebate size and patient out-of-pocket costs and whether this association differed by coverage type (ie, Medicare, commercial, or no insurance) and by period (ie, before and after 2014) in a nationally representative sample. Secondarily, we evaluated whether list prices were associated with the increase in out-of-pocket costs found with increasing rebate size.

## Methods

The Kaiser Permanente Washington Health Research Institute Institutional Review Board approved this study and determined that it was not human participants research and so informed consent was not necessary. The study is reported using the Strengthening the Reporting of Observational Studies in Epidemiology (STROBE) reporting guideline for cross-sectional studies.

### Data Sources

We used the 2007 to 2018 Household Component of the Medical Expenditure Panel Survey (MEPS) to obtain nsurance coverage and demographic information (including self-reported race to describe potential disparities) on surveyed individuals and the 2007 to 2018 MEPS Prescribed Medicines Files to obtain information on each prescription drug purchase made by these individuals. Advantages of the MEPS include its nationally representative sample of the US civilian noninstitutionalized population, including individuals without insurance. This survey also accurately measures out-of-pocket costs because it directly collects payment information from pharmacies, therefore excluding patient assistance programs (eg, copayment coupons) from out-of-pocket cost calculations (Steven Hill, PhD, Agency for Healthcare Research and Quality, email communication, June 2020).

Next, we obtained a 2007 to 2018 data set from SSR Health that contains quarterly list prices (ie, wholesale acquisition cost) and estimated net prices. These drug-specific net prices represent the revenue received per unit of drug by a manufacturer that reflects all price reductions, including rebates, discounts provided to 340B health care institutions, copayment coupons to patients, other purchase discounts, distribution fees, and product returns. SSR Health estimates these net prices quarterly for each drug by dividing drug-specific US revenues reported by the manufacturer by drug-specific US sales unit volume for all branded drugs sold to non-Medicaid health care payers by publicly listed companies.^18^ The data set contained drugs from more than 100 companies, accounting for more than 90% of US branded drug sales and has been used in multiple studies to document list and net price trends.^7,19,20^ For each branded drug, we calculated yearly list and net prices using the mean of quarterly prices weighted by sales volume.^7^

### Study Population

We included individuals who filled a branded drug of interest during a given year and who, for the entire year, were enrolled in Medicare or commercial insurance or were uninsured. Individuals simultaneously enrolled in Medicare and commercial plans (who most likely had commercial Medicare supplemental insurance) were categorized as having Medicare plans. Individuals covered under Medicaid (including those dually enrolled in Medicare and Medicaid) were excluded because out-of-pocket costs under Medicaid are typically small.

Drugs of interest were defined as single-source branded prescription drugs (ie, those without generic equivalents) observed in both the SSR Health and MEPS data sets in any observed quarter. We excluded branded drugs with generic equivalents because we expected rebates to be small for these drugs.^21^ For drugs with generic equivalents approved during the study period, we excluded the quarter before market exclusivity was lost and all subsequent quarters. This was done because prescription volume, and therefore rebate estimates, are less reliable during the transition from single-source branded drug to multisource branded drug.

### Variable of Interest

Our primary variable of interest was list price minus net price. For simplicity, we call this quantity “estimated rebates,” as in other studies,^19,20^ but this difference also includes other discounts, as noted in a previous section. An industry report^22^ indicated that rebates represented two-thirds of the difference between list and net price in 2018.

### Outcome Variable

Our outcome was out-of-pocket costs per branded prescription from MEPS. This represents the pharmacy-reported amount that an individual or family member paid for the medication and excludes payments made on their behalf, such as by manufacturers through copayment coupons.

### Covariate and Interaction Variables

To account for secular changes that may affect associations between rebates and out-of-pocket costs (eg, increasing use of coinsurance or high-deductible plan designs or increasing nonrebate discounts), we included categorical variables for each year (ie, year fixed effects). To draw inferences on the association between rebate size and out-of-pocket cost within drugs, we included drug-specific indicator variables (ie, drug fixed effects). We interacted coverage type (ie, Medicare, commercial, or no insurance) indicator variables with estimated rebates to allow for differential associations by coverage type in our overall analyses (eAppendix 1 in the Supplement).

Substantial changes occurred in insurance coverage around 2014, including Medicaid expansion, initiation of health insurance exchanges, a tax penalty for not having insurance (ie, the individual mandate), and the requirement that plans have annual drug out-of-pocket maximum limits.^23^ Several high-priced treatments for hepatitis C were also approved around 2014. We interacted an indicator variable for before and after 2014 with estimated rebates to evaluate differential associations by period.

### Statistical Analysis

#### All Models

We used the MEPS survey weights in all analyses to generate nationally representative estimates. Our unit of analysis was at the prescription level. All spending variables were adjusted to 2018 dollars using the US Consumer Price Index. All models adjusted for covariates described in the previous section. Robust standard errors were used to calculate 95% CIs, which were used to evaluate significance; results were considered significant if CIs did not cross 0. Analyses were conducted from August 2019 through March 2021 using Stata/MP statistical software version 15.1 (StataCorp).

#### Primary and Secondary Analyses

Our primary analyses evaluated the association between rebate sizes and out-of-pocket costs overall, by insurance coverage type, and before and after 2014. Associations were estimated using linear regression (eFigure 1 and eTable 1 [goodness-of-fit details] in the Supplement).^24,25^ We used recycled predictions to compare adjusted estimates of out-of-pocket costs based on observed rebate levels and no rebates (ie, if list price equaled net price) (eAppendix 2 in the Supplement). Secondarily, we evaluated whether rebates were associated out-of-pocket costs through list prices in 4 regressions. These evaluated associations between (1) rebates and list prices, (2) list prices and out-of-pocket costs controlling for rebates, (3) list prices and out-of-pocket costs not controlling for rebates; and (4) rebates and out-of-pocket costs controlling for list prices.

#### Sensitivity Analyses

We conducted 2 sensitivity analyses for our primary variable of interest (ie, rebate size) and 1 for our dependent variable (ie, out-of-pocket cost). Because net prices from SSR Health include nonrebate discounts, we also obtained net price estimates from the Federal Supply Schedule (FSS) from the Department of Veterans Affairs National Acquisition Center.^26^ These FSS prices represent the lower bound estimate of net prices paid by nonfederal payers. We then defined rebates as list price minus the greater of net or FSS prices. Second, PBMs use gross prices (ie, total amount paid to pharmacy by insurer, patient, and all other sources) to calculate out-of-pocket costs for prescription fills that are subject to deductibles or coinsurance.^27^ As a second sensitivity analysis, we calculated rebates as gross prices from MEPS minus net price.^7,28^ Third, because MEPS improved the quality of out-of-pocket cost data starting in 2009,^29^ a sensitivity analysis excluded all data before 2009. As a data check, we replicated list price and net price trends from a 2020 descriptive study^7^ that used SSR Health data (eFigure 2 in the Supplement).

Additionally, rebates are expected to affect only individuals subject to cost sharing (eg, individuals in plans that cover all out-of-pocket costs for drugs would not be affected by rising rebates). Therefore, as a further sensitivity analysis, we estimated the association between rebates and out-of-pocket costs by calculating the probability of incurring any out-of-pocket costs for a branded prescription fill using a binomial distribution and logit link. We then estimated the increase in out-of-pocket costs associated with rebates among prescriptions with nonzero out-of-pocket costs by log-transforming rebates and out-of-pocket costs and then evaluating the association using linear regression.

## Results

### Descriptive Population Characteristics

**Figure 2.**
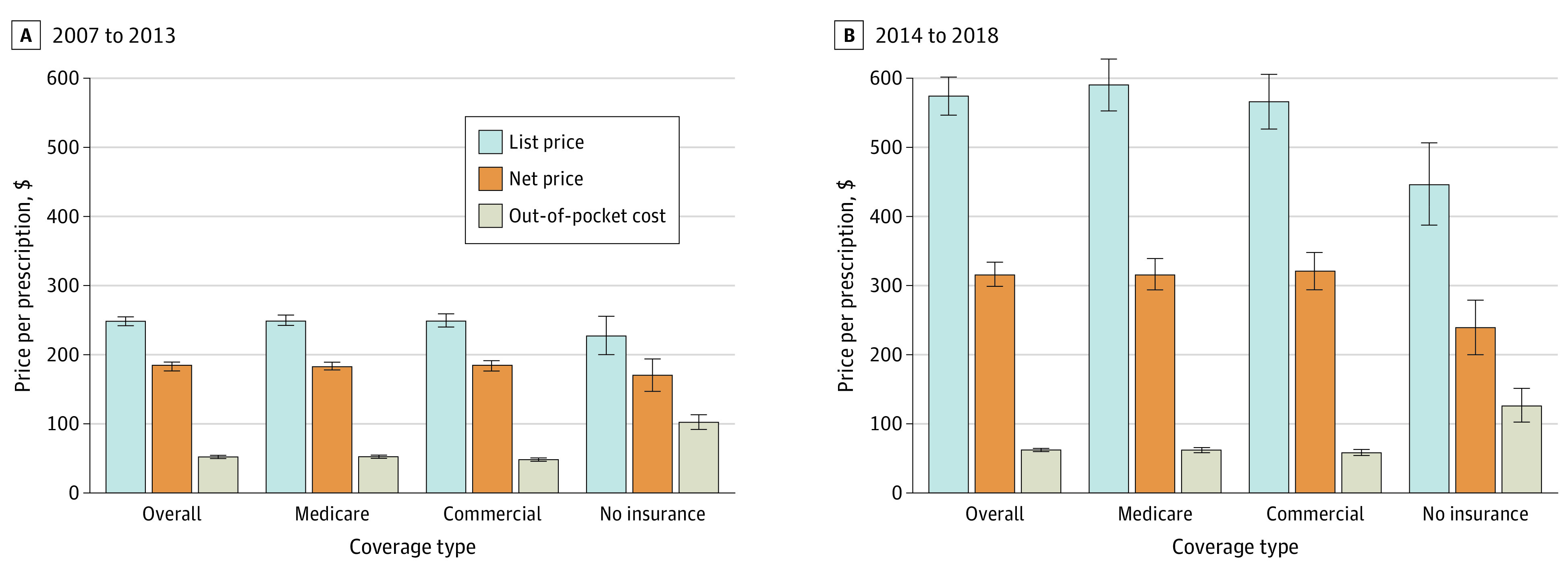
Mean List Price, Net Price, and Out-of-Pocket Cost by Coverage Type Amounts are per branded prescription in 2018 US dollars. Whiskers indicate 95% CIs.

Among 38 131 individuals, the mean age was 54 years (95% CI, 54-55 years), with 22 044 women (57.8%) and 29 086 White individuals (76.3%). Reflecting the inclusion criteria of individuals using single-source branded drugs, our sample included a larger proportion of individuals with Medicare insurance and fewer individuals without insurance. For example, among 13 024 individuals in our sample from 2014 to 2018, compared with the 2018 general US population, 5773 individuals (44.3%) vs 13.8% of individuals had Medicare insurance and 672 individuals (5.2%) vs 8.9% of individuals had no insurance (Table 1).^30,31^ Individuals with Medicare insurance made the most branded prescription purchases, while individuals who were uninsured made the fewest such purchases. Individuals who were uninsured were younger, in poorer health, and more likely to be members of a racial minority group and had lower personal income compared with our overall sample (eTable 2 in the Supplement). The mean annual personal income among individuals without insurance was $19 351 (95% CI, $17 537-$21 165) in 2007 to 2013 and $22 628 (95% CI, $18 902-$26354) in 2014 to 2018, while the mean individual income for the overall sample was $37 575 (95% CI, $36 630-$38 520) in 2007 to 2013 and $42 207 ($41 018-$43 395) in 2014 to 2018. Individuals with Medicare and commercial plans used drugs with higher list and net prices but had half the out-of-pocket costs per prescription compared with uninsured individuals (Figure 2). The population characteristics from 2007 to 2013 were similar; however, there was a lower percentage of individuals without insurance and a higher percentage of individuals with Medicare compared with 2014 to 2018.

**Table 1.** Characteristics of Individuals Included in the Sample

	2007-2013	2014-2018
Total sample size, No.		
Survey-weighted person-years in 1000s	435 565	193 699
Unweighted unique persons^a^	26 241	13 024
Characteristic, unweighted No. (%)		
Age category, y		
≤18	1864 (7.1)	960 (7.4)
19-35	2363 (9.0)	1291 (9.9)
36-64	9921 (37.8)	5720 (43.9)
≥65	12 093 (46.1)	5053 (38.8)
Sex		
Women	15 168 (57.8)	7504 (57.6)
Men	11 073 (42.2)	5520 (42.4)
Race		
White only	20 011 (76.3)	9908 (76.1)
Black only	4123 (15.7)	1986 (15.2)
Other^b^	2107 (8.0)	1130 (8.7)
Poor health at any time within year	2736 (10.4)	1445 (11.1)
Individual personal income, mean (95% CI), $	37 575 (36 630-38 520)	42 207 (41 018-43 395)
Not employed at any time within year	11 648 (44.4)	6541 (50.2)
4 y of college education	4066 (15.5)	2210 (17.0)
Insurance coverage type^c^		
Medicare coverage entire year	8897 (33.9)	5773 (44.3)
Commercial coverage entire year	15 086 (57.5)	6645 (51)
Uninsured entire year	2468 (9.4)	672 (5.2)
Single-source branded drug purchases per year, mean (95% CI)	8 (8-8)	6 (6-6)

^a^ The total number of unique persons is 38 131 because a small number of individuals are observed in both 2013 and 2014.

^b^ Other includes Native American or Native Alaskan, Asian, and Native Hawaiian or Pacific Islander individuals and individuals with multiple races.

^c^ A small number of unique persons changed between Medicare, Commercial, or uninsured status between years.

### Descriptive Drug Price Trends

**Figure 3.**
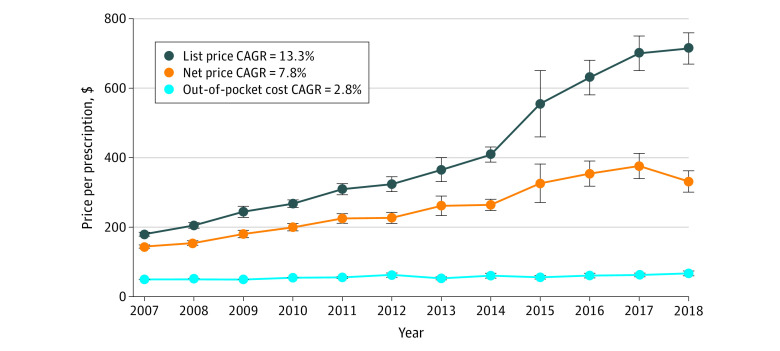
Trends in List Price, Net Price, and Out-of-Pocket Cost Per Branded Prescription Amounts are in 2018 US dollars. CAGR indicates compound annual growth rate; whiskers, 95% CIs.

Our sample from 2007 to 2018 included a total of 444 unique single-source branded drugs with a survey-weighted 4.7 billion prescriptions (Figure 3; eTable 3 in the Supplement). All observed price growth outpaced inflation; from 2007 to 2018, mean (SE) list prices per prescription increased from $180 ($3) to $714 ($23), for an annual growth rate of 13.3%; net prices from $145 ($2) to $332 ($16), for an annual growth rate of 7.8%; and out-of-pocket costs from $50 ($1) to $67 ($4), for an annual growth rate of 2.8%. Estimated mean (SE) rebates grew the most rapidly, from $34 ($1) per prescription in 2007 to $374 ($9) per prescription in 2018, for a growth rate of 24.4% per year, matching a Medicare Trustee report of substantially rising rebates.^4^ Mean (SE) estimated rebates ranged from $17 ($0)for hypothyroid treatments to $11 567 ($1869) for hepatitis C treatments (eTable 4 in the Supplement).

### Primary and Secondary Analyses

We found that increased estimated rebates were associated with an overall increase in out-of-pocket costs of $4 (95% CI, $4-$4) per branded prescription in 2007 to 2013 and $11 per branded prescription (95% CI, $10-$12) in 2014 to 2018 (Table 2; eTable 5 in the Supplement). This association varied by coverage type. For instance, among individuals with Medicare or commercial insurance or who were uninsured, estimated rebates were associated with increased out-of-pocket costs in 2014 to 2018 of $13 (95% CI, $12-$13), $6 (95% CI, $6-$7), and $39 (95% CI, $34-$44), respectively. Because individuals could fill more than 1 prescription per year, we also calculated total out-of-pocket costs per branded drug user per year for Medicare or commercial insurance or uninsured status in 2014 to 2018, as $85 (95% CI, $79-$91), $34 (95% CI, $32-$37), and $211 (95% CI, $178-$243), respectively (eTable 6 in the Supplement). For individuals without insurance, increased out-of-pocket costs associated with rebates consumed approximately 1% of their annual personal income in both periods (1.1% in 2007-2013 and 0.9% in 2014-2018) because the increase in out-of-pocket costs per user per year in 2007 to 2013 was $210 (95% CI, $185-$234) for these individuals.

**Table 2.** Mean Out-of-Pocket Costs per Branded Prescription

	Out-of-pocket costs per prescription (95% CI), 2018 $^a^		
	With observed rebates^b^	With no rebates^c^	Difference
**2007-2013**			
Overall	52 (51-53)	48 (47-48)	4 (4-4)
By coverage type			
Medicare	52 (51-53)	45 (44-46)	7 (7-7)
Commercial	48 (47-48)	48 (47-49)	0 (0-0)
Uninsured	103 (95-110)	71 (65-77)	32 (29-35)
**2014-2018**			
Overall	61 (60-62)	50 (49-51)	11 (10-12)
By coverage type			
Medicare	61 (60-62)	48 (47-49)	13 (12-13)
Commercial	57 (56-59)	51 (49-53)	6 (6-7)
Uninsured	126 (114-137)	87 (77-97)	39 (34-44)

^a^ Models are adjusted for year and drug and include interactions with coverage type and indicators for 2007 to 2013 and 2014 to 2018.

^b^ These are factual estimates: the model-adjusted estimate of out-of-pocket costs with observed rebates.

^c^ These are counterfactual estimates: the model-adjusted estimate of out-of-pocket costs if rebates were set to zero.

In our secondary analyses, we found that a $1 increase in rebate size was associated with an increase in list price per branded prescription of $2.75 (95% CI, $2.55-$2.96) from 2007 to 2013 and $2.09 (95% CI, $1.96-$2.22) from 2014 to 2018 (eTable 7 in the Supplement). In turn, a $1 increase in list price was associated with an increase in out-of-pocket costs per branded prescription of $0.02 (95% CI, $0.01-$0.03) from 2007 to 2013 and from 2014 to 2018. Controlling for list prices, there was no association between rebates and out-of-pocket costs after controlling for list prices. From 2014 to 2018, the change was −$0.01 (95% CI, −$0.04 to $0.02).

### Sensitivity Analyses

Sensitivity analyses using alternative rebate definitions or excluding pre-2009 data yielded similar results (eTable 8 in the Supplement). Additionally, we found that increased rebates were not associated with increased probability of incurring out-of-pocket costs. However, among prescriptions with nonzero out-of-pocket costs across all insurance types, a 1% increase in estimated rebates was associated with a 0.32% increase in out-of-pocket costs (95% CI, 0.31%-0.35%) in 2007 to 2013 and a 0.19% increase in out-of-pocket costs (95% CI, 0.16%-0.22%) in 2014 to 2018 (eTable 9 in the Supplement).

## Discussion

This cross-sectional study found that for single-source branded drugs, list prices, net prices, out-of-pocket costs, and estimated rebates all outpaced inflation from 2007 to 2018, with rebates growing the most rapidly. Rebate increases were associated with mean out-of-pocket cost increases per prescription of $11 in 2014 to 2018. However, increases were as high as $39 for individuals without insurance, with $13 and $6 increases for those with Medicare or commercial insurance, respectively. Studies from 2004^32^ and 2003^33^ found that $10 to $20 cost-sharing increases are associated with decreased medication adherence. Among individuals with lower incomes and among older adults, increasing prescription cost sharing can be associated with increased emergency department use, more frequent hospitalizations, and other poor health outcomes.^15,16,34,35^

We also found that the increases in out-of-pocket costs associated with rebates were greater at later time periods. This increase may be associated with the substantial growth in rebates and the increasing use of cost sharing (ie, high-deductible and coinsurance plans) that ties out-of-pocket costs to list prices. Furthermore, our secondary analyses indicated that the positive association of rebates with out-of-pocket costs was associated with simultaneous increases in list prices. Larger rebates without list price increases should benefit enrollees through lower insurer spending and potentially lower premiums without increasing out-of-pocket costs for individuals actually filling prescriptions. Our results indicated that when list prices were constant, increased rebates were not associated with increased patient out-of-pocket costs.

Our findings have health equity implications. Rebates were associated with increased out-of-pocket costs that were more than 3-fold greater among individuals without insurance than among individuals in the overall sample in 2014 to 2018 (Table 2). This adds to the financial burden of individuals without insurance, who already had the lowest income levels. Indeed, increased out-of-pocket costs associated with rebates consumed 1% of their annual personal income in both periods. This disparity may also have clinical implications. Individuals without insurance in our sample had the poorest health, and individuals with lower incomes are less likely to adhere to treatments when out-of-pocket costs increase.^36^ Our findings, combined with the probability that individuals with worse health status require more intensive pharmacologic treatment, suggest a potential disproportional clinical outcome associated with increasing rebates among individuals without insurance. Furthermore, individuals without insurance were more likely to be in racial minority groups, which may amplify preexisting disparities in health care access.^37,38^ Additionally, whereas individuals with insurance benefit from increasing rebates (through decreased future premiums),^39^ individuals without insurance do not.

We also found that the increases in out-of-pocket costs associated with larger rebates were greater for individuals covered by Medicare than for those covered by commercial insurance. This could be because coinsurance is more commonly used in Medicare plans and because Medicare Part D plans typically do not have annual out-of-pocket maximums.^40^ Indeed, findings from Trish et al^40^ suggest that increases in list prices may have contributed to the increased numbers of individuals in Medicare who reached catastrophic out-of-pocket spending levels. Nevertheless, the increase in costs associated with rebates also grew among individuals covered by commercial insurance, potentially due to increased uptake of high-deductible health plans among this population.^41^ This outcome suggests that rebates and increases in list prices may be associated with negative outcomes among individuals with commercial insurance in coming years.

Overall, these results suggest that manufacturers may have perverse incentives to increase list prices in order to offer larger rebates. While this may partly dampen the growth in insurer costs and overall premiums, it is associated with increased out-of-pocket costs for patients filling prescriptions, especially for individuals without insurance. The uninsured population, while relatively small, is of high concern for the US public.^42^ Furthermore, this population has been growing in recent years, and the COVID-19 pandemic may add to the number of individuals who lose employer-based insurance coverage and become newly uninsured and exposed to list prices.^43^ This situation highlights the need for affordable insurance options, such as through Medicaid expansion, which decreases health care-related financial distress.^44^ Federal proposals to share rebates at the point of sale with patients enrolled in Medicare Part D raise concerns about increasing premiums, in part because changing rebate strategies does not guarantee equivalent list price reductions from manufacturers.^5,45,46^ Another alternative is encouraging plans to use copayments (ie, fixed dollar amounts per prescription) rather than coinsurance, thereby decoupling out-of-pocket costs from list prices.

### Limitations

Our study has several limitations. First, our results do not necessarily generalize to drugs used to treat rare conditions because MEPS masks the identity of drugs used by fewer than 200 000 people per year. These drugs typically have smaller rebates. Second, our results do not generalize to the fewer than 10% of branded drugs marketed by nonpublicly traded companies, which are not in the SSR Health data set. Third, while SSR Health net price estimates are increasingly applied in research^7,19,20^ and in determining value-based drug prices,^47^ it is not known how well the data truly represent net prices paid by PBMs. These rebate estimates may also be less precise for specific drug classes.^48^ We therefore focused our analyses on overall associations across classes and are further reassured that sensitivity analyses estimating rebates from alternative data sources yielded similar conclusions. Fourth, our rebate estimates were means across coverage types, but rebates may vary across coverage types. However, commercial rebates are expected to be within 10% of Medicare rebates, according to an industry report.^49^ Fifth, our analyses focused on filled prescriptions and did not address that patients who could not afford out-of-pocket costs may not have filled their prescriptions.

## Conclusions

This study found that rebates for single-source branded drugs increased substantially over the past 12 years. We found that rebates, likely through simultaneous increases in list prices, were associated with out-of-pocket cost increases and that these impacts appear to have grown. This system amplifies health equity concerns, as individuals without insurance are disproportionately disadvantaged. These findings suggest that with the number of individuals without insurance expected to increase, future research and policy considerations should focus on potential solutions to decouple list prices and out-of-pocket costs, especially for individuals without insurance.

## Notes

Supplement. **eAppendix 1.** Analytic Model Specification **eAppendix 2.** Generating Estimates Using the Method of Recycled Predictions **eFigure 1.** Goodness-of-Fit Modified Hosmer-Lemeshow Plots of Mean Residuals by 20 Quantiles Model-Predicted Out-of-Pocket Costs for 3 Model Specifications **eFigure 2.** Replicating Hernandez et al Price Trends, 2007-2018 **eFigure 3.** Trends in List, Net, Gross, and Federal Supply Schedule Price and Out-of-Pocket Cost Per Prescription, 2007-2018 **eTable 1.** Pearson Correlation of Residual and Predicted Out-of-Pocket Costs for 3 Model Specifications **eTable 2.** Characteristics of Individuals Included in the Sample by Coverage Type, 2007-2013 and 2014-2018 (Survey-Weighted Values) **eTable 3.** Number of Unique Drugs and Survey-Weighted Prescriptions by Year **eTable 4.** Out-of-Pocket Costs, List Price, Net Price, and Estimated Rebates by Drug Class, 2007-2018 **eTable 5.** Regression Coefficients From Primary Analysis, Overall Model, 2007-2013 and 2014-2018 **eTable 6.** Total Out-of-Pocket Costs for Single-Source Branded Prescriptions Per Branded Drug User Per Year **eTable 7.** Estimated Change in Dependent Variable Given $1 Increase in Independent Variable, 2007-2013 and 2014-2018 **eTable 8.** Sensitivity Analyses of Estimated Out-Of-Pocket Costs With 2009-2013 Preperiod and Alternative Rebate Definitions **eTable 9.** Association of Rebates With Probability of Out-of-Pocket Costs and Increase in Out-of-Pocket Costs eReferences
