# Supplementary material for: Association of Branded Prescription Drug Rebate Size and Patient Out-of-Pocket Costs in a Nationally Representative Sample, 2007-2018
Source: JAMA Netw Open. 2021 Jun 14;4(6):e2113393. doi: 10.1001/jamanetworkopen.2021.13393 (PMC8204201; doi:10.1001/jamanetworkopen.2021.13393)
Supplement: Supplement. — eAppendix 1. Analytic Model Specification eAppendix 2. Generating Estimates Using the Method of Recycled Predictions eFigure 1. Goodness-of-Fit Modified Hosmer-Lemeshow Plots of Mean Residuals by 20 Quantiles Model-Predicted Out-of-Pocket Costs for 3 Model Specifications eFigure 2. Replicating Hernandez et al Price Trends, 2007-2018 eFigure 3. Trends in List, Net, Gross, and Federal Supply Schedule Price and Out-of-Pocket Cost Per Prescription, 2007-2018 eTable 1. Pearson Correlation of Residual and Predicted Out-of-Pocket Costs for 3 Model Specifications eTable 2. Characteristics of Individuals Included in the Sample by Coverage Type, 2007-2013 and 2014-2018 (Survey-Weighted Values) eTable 3. Number of Unique Drugs and Survey-Weighted Prescriptions by Year eTable 4. Out-of-Pocket Costs, List Price, Net Price, and Estimated Rebates by Drug Class, 2007-2018 eTable 5. Regression Coefficients From Primary Analysis, Overall Model, 2007-2013 and 2014-2018 eTable 6. Total Out-of-Pocket Costs for Single-Source Branded Prescriptions Per Branded Drug User Per Year eTable 7. Estimated Change in Dependent Variable Given $1 Increase in Independent Variable, 2007-2013 and 2014-2018 eTable 8. Sensitivity Analyses of Estimated Out-Of-Pocket Costs With 2009-2013 Preperiod and Alternative Rebate Definitions eTable 9. Association of Rebates With Probability of Out-of-Pocket Costs and Increase in Out-of-Pocket Costs eReferences [file jamanetwopen-e2113393-s001.pdf]

Supplemental Online Content

Yeung K, Dusetzina SB, Basu A. Association of branded prescription drug rebate size and patient out-of-pocket costs in a nationally representative sample, 2007-2018. *JAMA Netw Open*. 2021;4(6):e2113393. doi:10.1001/jamanetworkopen.2021.13393

eAppendix 1. Analytic Model Specification

eAppendix 2. Generating Estimates Using the Method of Recycled Predictions

eFigure 1. Goodness-of-Fit Modified Hosmer-Lemeshow Plots of Mean Residuals by 20 Quantiles Model-Predicted Out-of-Pocket Costs for 3 Model Specifications

eFigure 2. Replicating Hernandez et al Price Trends, 2007-2018

eFigure 3. Trends in List, Net, Gross, and Federal Supply Schedule Price and Out-of-Pocket Cost Per Prescription, 2007-2018

eTable 1. Pearson Correlation of Residual and Predicted Out-of-Pocket Costs for 3 Model Specifications

eTable 2. Characteristics of Individuals Included in the Sample by Coverage Type, 2007-2013 and 2014-2018 (Survey-Weighted Values)

eTable 3. Number of Unique Drugs and Survey-Weighted Prescriptions by Year

eTable 4. Out-of-Pocket Costs, List Price, Net Price, and Estimated Rebates by Drug Class, 2007-2018

eTable 5. Regression Coefficients From Primary Analysis, Overall Model, 2007-2013 and 2014-2018

eTable 6. Total Out-of-Pocket Costs for Single-Source Branded Prescriptions Per Branded Drug User Per Year

eTable 7. Estimated Change in Dependent Variable Given $1 Increase in Independent Variable, 2007-2013 and 2014-2018

eTable 8. Sensitivity Analyses of Estimated Out-Of-Pocket Costs With 2009-2013 Preperiod and Alternative Rebate Definitions

eTable 9. Association of Rebates With Probability of Out-of-Pocket Costs and Increase in Out-of-Pocket Costs

eReferences

This supplemental material has been provided by the authors to give readers additional information about their work.

© 2021 Yeung K et al. *JAMA Network Open.*

eAppendix 1. Analytic Model Specification

To generate our overall estimates, we used a linear regression model according to the following specification:

$$E[Y_{icdt}| \cdot]) = \beta_0 + \beta_1\, rebate_{idt} + \beta_2\, cov\_type_c + \beta_3 post_t + \beta_4 year_t + \beta_5 drug_d + \beta_6\, rebate_{idt} * cov\_type_c + \beta_7\, rebate_{idt} * post_t + \beta_8\, cov\_type_c * post + \beta_9\, rebate_{idt} * cov\_type_c * post + \alpha X_i$$

Where:

Y_{icdt}= Out-of-pocket costs in 2018 US dollars for prescription fill “i” for drug “d” in year “t”

$Rebate_{idt}$ = Estimated rebate size in 2018 US dollars for prescription fill “i” for drug “d” in year “t”

cov_type_c = Categorical variable for Medicare coverage entire year, commercial coverage entire year or uninsured entire year

$post_t$ = Indicator variable for 2007-2013 or 2014-2018

$year_t$ = Categorical variable for each of 11 years

$drug_d$ = Categorical variable for each of the single-source branded drugs in our sample

To generate our estimates by coverage type, we used the same model specification, except that we excluded the “cov_type_c” term and repeated the regressions in each of the 3 subpopulations (Medicare, Commercial, and Uninsured).

© 2021 Yeung K et al. *JAMA Network Open.*

eAppendix 2. Generating Estimates Using the Method of Recycled Predictions

Overview: The parameter of interest is the average association between rebates and out-of-pocket costs (reported in "Difference" column in Table 2 of our main manuscript). This parameter is informed by the coefficients representing the rebate term with the coverage type and post interactions. Specifically, these terms are:

$$\beta_1\, rebate_{idt} + \beta_6\, rebate_{idt} * cov\_type_c + \beta_7\, rebate_{idt} * post_t + \beta_9\, rebate_{idt} * cov\_type_c * post$$

These terms and all regression coefficients are estimated and applied in the following steps:

1. Conduct regression: Using the model specification above and as described in the "Primary and Secondary Analyses" section of the main manuscript text.

2. Generate estimate of out-of-pocket costs with observed rebates (i.e., factual estimate): Using the regression model, we predict the out-of-pocket costs for each observation. Then we take the mean of the predicted out-of-pocket costs for those observations. This adjusted mean estimate is reported in "With Observed Rebates" column in Table 2 of our main manuscript.

3. Generate estimate of out-of-pocket costs if rebates were set to zero (i.e., counterfactual estimate): This estimate is also known as the adjusted mean out-of-pocket estimate if list prices were equal to net prices (i.e., if there were no rebates). To generate this estimate, we use the same procedure as step #2 above, except that after conducting the regression, we first set the values of variables representing the rebate equal to zero for each observation, so those coefficients no longer contribute to the predicted values (i.e., "turning off" β_1, β_6,β_7,β_9). We then predict out-of-pocket costs for each observation. Then, we take the mean of the predicted out-of-pocket costs for these observations. This adjusted mean estimate is reported in "With Zero Rebates" column in Table 2 of our main manuscript.

4. Generating association between rebates and out-of-pocket costs: The difference between the counterfactual and factual estimates represent the adjusted mean association between rebates and out-of-pocket costs which is reported in the "Difference" column in Table 2 of our main manuscript.

© 2021 Yeung K et al. *JAMA Network Open.*

eFigure 1. Goodness-of-Fit Modified Hosmer-Lemeshow Plots of Mean Residuals by 20 Quantiles Model-Predicted Out-of-Pocket Costs for 3 Model Specifications

eFigure 1a. Plot based on linear regression of raw-scale (i.e., untransformed) out-of-pocket costs.

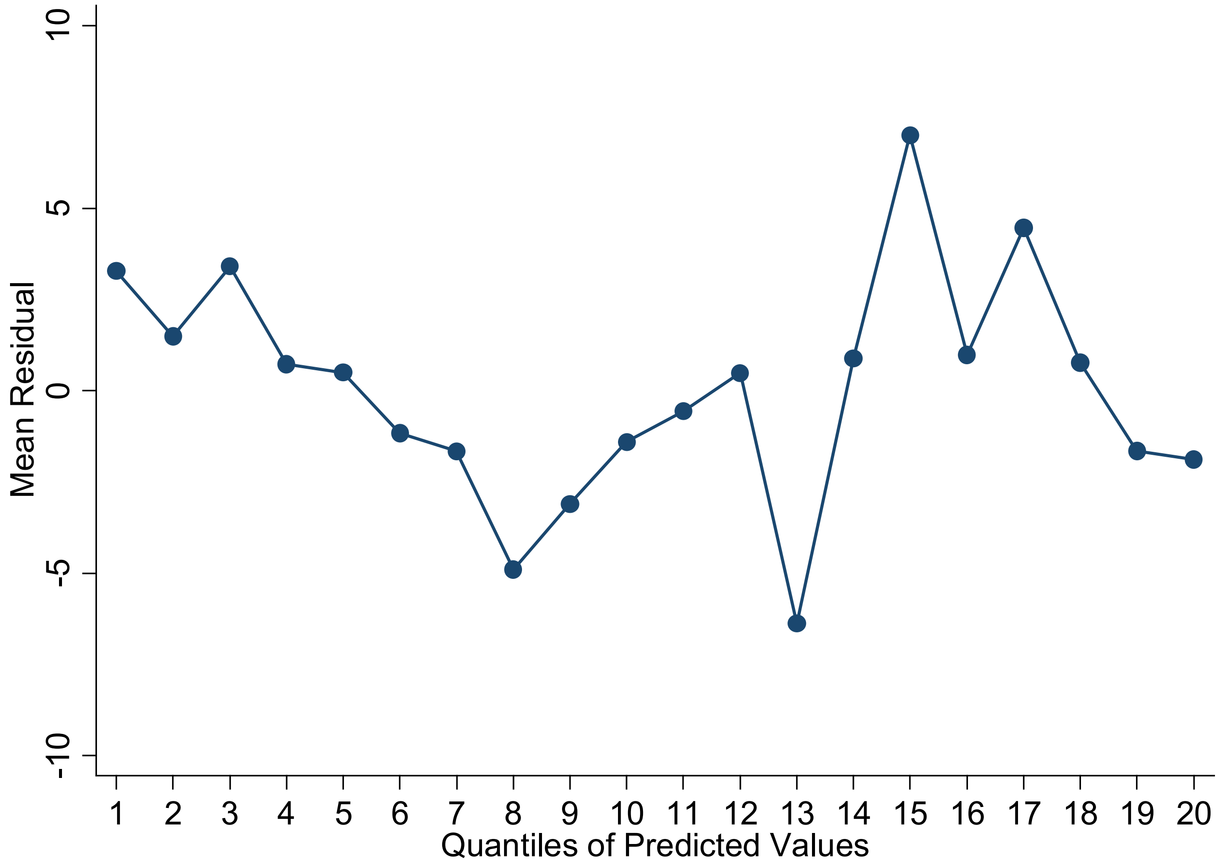

© 2021 Yeung K et al. *JAMA Network Open.*

eFigure 1b. Plot based on linear regression of logged out-of-pocket costs.

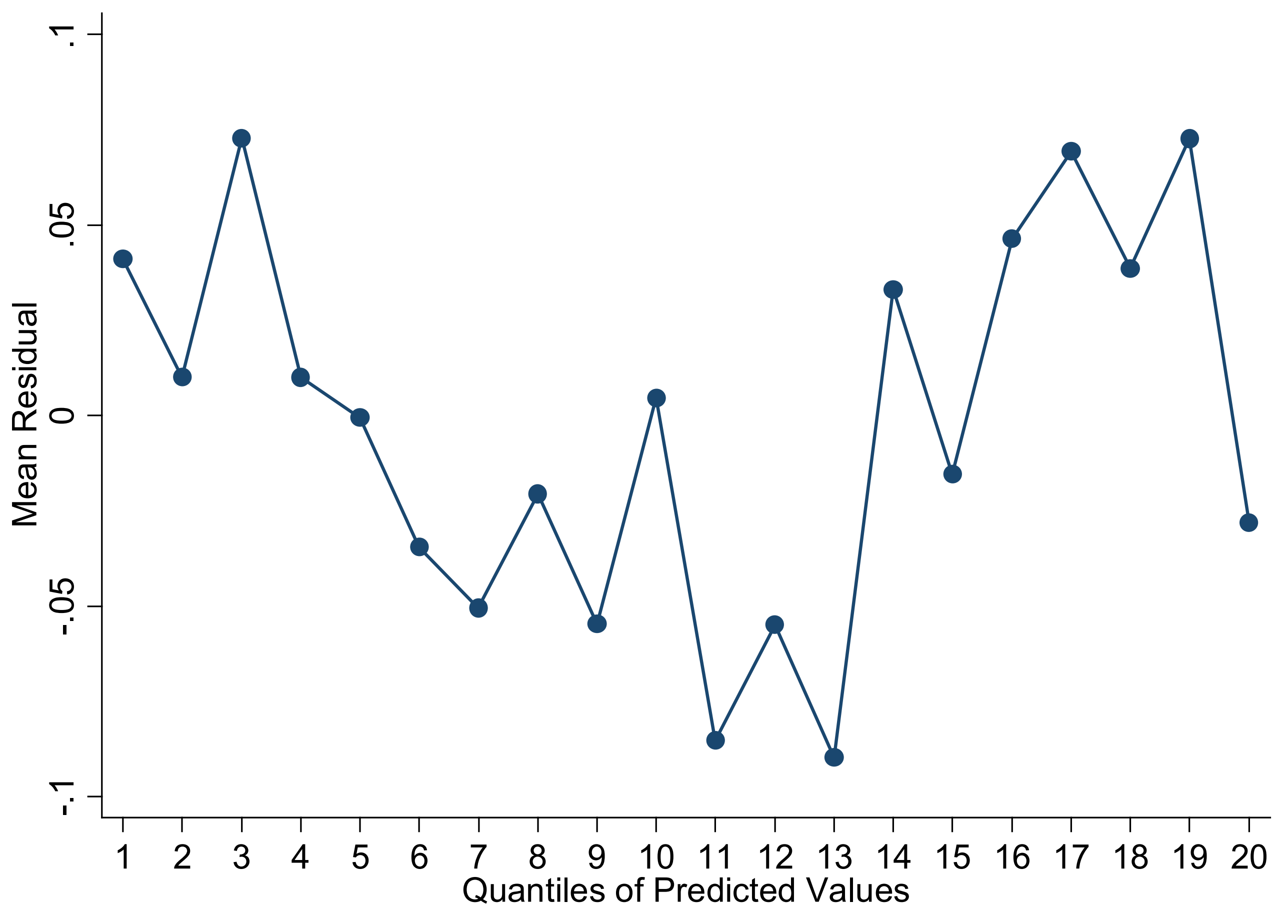

© 2021 Yeung K et al. *JAMA Network Open.*

eFigure 2. Replicating Hernandez et al Price Trends, 2007-2018

We were able to replicate Hernandez et al.'s list and net trends using the methods they describe. Our trends in Figure 3 agree with that study, with list and net prices increasing over time. However, due to differences in inclusion criteria, our observed trends grew more rapidly. Since our focus was on the association of rebates with out-of-pocket costs, we excluded multi-source branded drugs (i.e., drugs with generic equivalents) because they have low rebates. Further, Hernandez et al. excluded drugs that entered the market after 2007. In addition, our study included only drugs observed in MEPS, which masks the identity of drugs used by fewer than 200,000 people, which typically have smaller rebates.

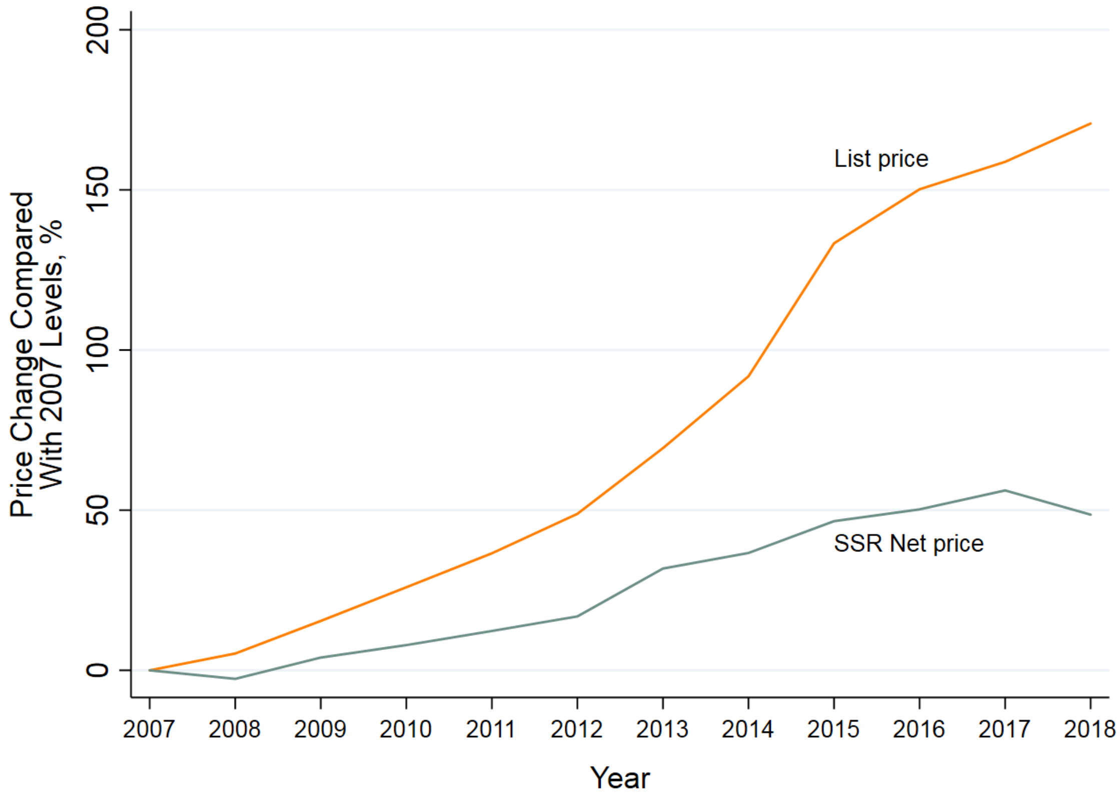

© 2021 Yeung K et al. *JAMA Network Open.*

eFigure 3. Trends in List, Net, Gross, and Federal Supply Schedule Price and Out-of-Pocket Cost Per Prescription, 2007-2018

CAGR, compound annual growth rate.

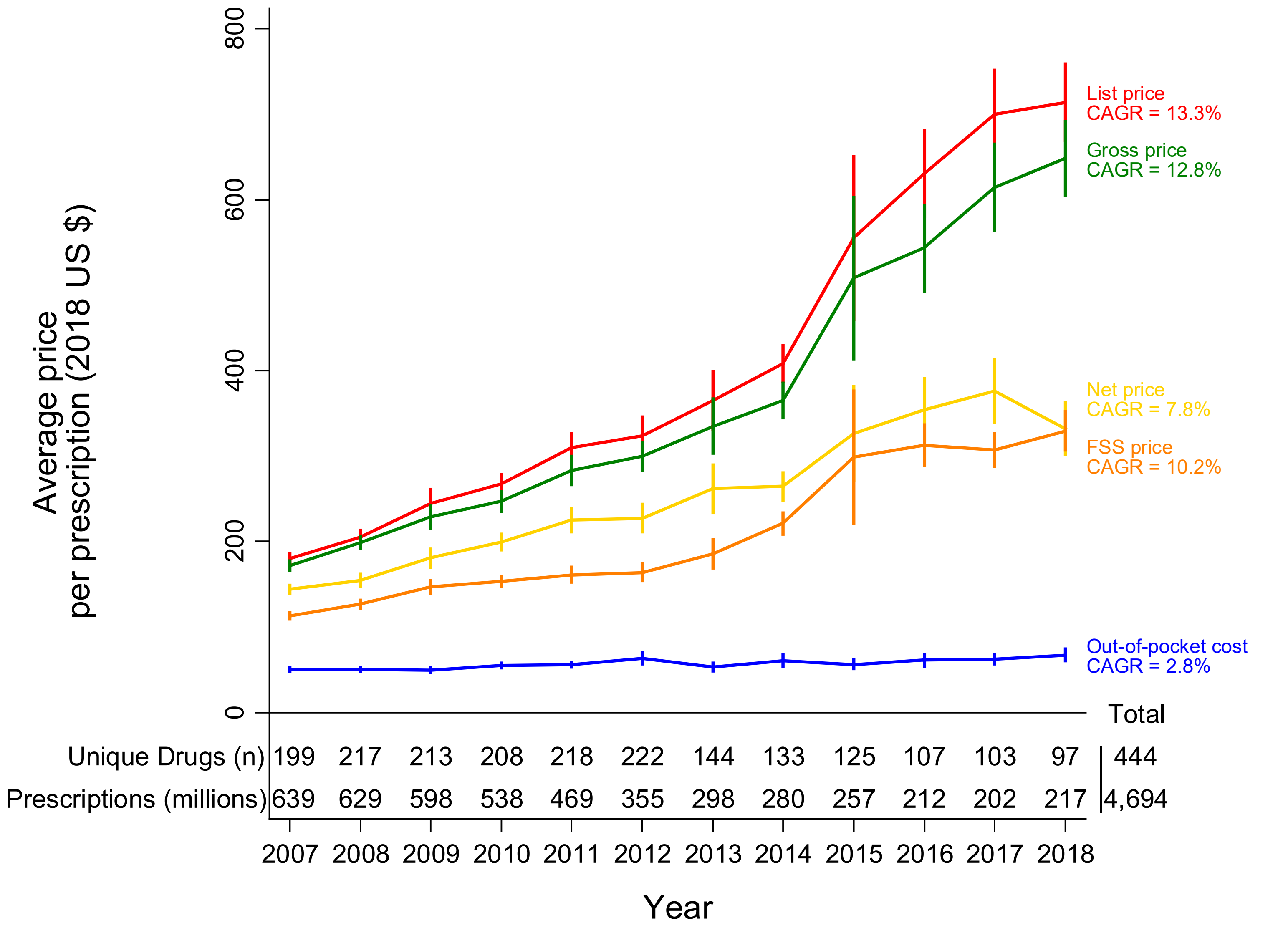

© 2021 Yeung K et al. *JAMA Network Open.*

eTable 1. Pearson Correlation of Residual and Predicted Out-of-Pocket Costs for 3 Model Specifications

**Model**	**Pearson's correlation**
Untransformed estimated rebate and out-of-pocket cost with linear regression	-0.0093
Logged out-of-pocket and logged estimated rebate with linear regression	-0.0010

© 2021 Yeung K et al. *JAMA Network Open.*

eTable 2. Characteristics of Individuals Included in the Sample by Coverage Type, 2007-2013 and 2014-2018 (Survey-Weighted Values)[a]

**Coverage type**	**Medicare**		**Commercial**		**Uninsured**	
Years	2007-2013	2014-2018	2007-2013	2014-2018	2007-2013	2014-2018
Survey-weighted person-years in 1000s[b]	149,700	82,064	260,369	105,358	25,496	6,276
Unweighted unique persons[b]	8,897	5,773	15,086	6,645	2,468	672
Age category						
≤18	0%	0%	13%	13%	7%	7%
19-35	0%	0%	15%	18%	20%	17%
36-64	10%	12%	70%	66%	72%	74%
≥65	89%	88%	2%	3%	1%	2%
Female sex	57%	57%	56%	58%	57%	54%
Race						
White only	87%	85%	86%	84%	81%	76%
Black only	8%	9%	8%	9%	12%	14%
Other	4%	6%	6%	8%	6%	9%
Poor health at sometime within year	15%	16%	5%	5%	18%	16%
Individual personal income ($), 95%CI	30528 (29214 to 31842)	35893 (34481 to 37305)	43502 (42442 to 44561)	48291 (46685 to 49896)	19351 (17537 to 21165)	22628 (18902 to 26354)
Not employed at sometime within year	86%	87%	20%	19%	48%	44%
4 years of college education	14%	15%	22%	23%	11%	12%
Medicare coverage entire year	100%	100%	0%	0%	0%	0%
Commercial coverage entire year	44%	43%	100%	100%	0%	0%
Uninsured entire year	0%	0%	0%	0%	100%	100%
Total included branded prescriptions filled per year (n)	10	6.8	7	5.5	6.5	5.4

[a]Reported percentages are survey-weighted values.

[b]There are slightly fewer individuals overall than the sum of individuals by coverage type since a few individuals switched coverage type between survey years.

© 2021 Yeung K et al. *JAMA Network Open.*

eTable 3. Number of Unique Drugs and Survey-Weighted Prescriptions by Year

Year	2007	2008	2009	2010	2011	2012	2013	2014	2015	2016	2017	2018	Total
Unique Drugs (n)	199	217	213	208	218	222	144	133	125	107	103	97	444
Prescriptions (millions)	639	629	598	538	469	355	298	280	257	212	202	217	4,694

© 2021 Yeung K et al. *JAMA Network Open.*

eTable 4. Out-of-Pocket Costs, List Price, Net Price, and Estimated Rebates by Drug Class, 2007-2018							
**Drug Class**	**Number of Unique Drugs (n)**	**Number of prescriptions (survey weighted)**	**Out-of-pocket costs**	**List price**	**Net Price**	**Estimated Rebates**	**Estimated Rebates as percent of list price**
			2018 US dollars				
ADHD treatments	8	81,356,085	56	249	171	78	31
Acne treatments (Topical)	3	2,497,894	42	440	248	192	44
Alzheimer's disease treatments	5	27,139,614	80	389	336	53	14
Anemia treatments (B12 deficiency)	1	422,612	27	1099	894	205	19
Anti-Ulcer (Other)	1	1,391,766	48	291	272	19	7
Anti-Ulcer (PPIs)	6	215,648,725	51	328	132	196	60
Antibacterials	1	167,492	129	527	345	182	35
Anticonvulsants	11	37,274,236	64	387	316	70	18
Antiemetics	1	528,420	87	483	435	47	10
Antifungals	2	308,121	28	2898	2481	417	14
Antiviral (Other)	4	20,910,640	55	220	156	64	29
Atopic dermatitis	1	874,110	44	195	119	76	39
Atypical antipsychotics	10	64,324,361	78	627	544	83	13
Alpha blockers	2	23,656,833	54	200	168	32	16
Benzodiazepines	2	2,629,709	37	180	145	35	19
Bowel preparation	1	1,824,359	40	68	43	25	37
COPD treatments (Bronchodilators)	10	194,011,928	29	91	38	53	58
COPD treatments (Combos)	5	247,956,683	57	403	233	170	42
COPD treatments (Glucocorticoids)	6	73,855,481	51	552	372	180	33
COPD/Asthma treatments (Other)	3	93,608,822	49	217	173	44	20
Cardiovascular (ARBs, ARB combos)	6	249,905,585	49	149	100	49	33
Cardiovascular (Beta blockers)	3	107,217,488	38	124	88	36	29
Cardiovascular (Other)	2	13,033,482	57	420	339	80	19
Chelating agents	1	3,038,386	97	1321	878	442	33

© 2021 Yeung K et al. *JAMA Network Open.*

Cholesterol (Non-PCSK9)	10	704,266,418	52	223	147	76	34
Contraceptives	9	86,675,831	32	116	86	30	26
DMARDs (Anti-TNF)	4	36,030,628	307	3947	3104	842	21
Depression	12	215,747,316	52	211	175	36	17
Diabetes (DPP-4 inhibitors)	6	110,652,004	57	494	256	238	48
Diabetes (GLP-1 agonists)	5	41,971,377	75	679	457	222	33
Diabetes (Long-acting insulins)	5	188,272,140	59	503	263	240	48
Diabetes (Other)	4	8,008,472	31	240	150	91	38
Diabetes (Rapid-acting/mix insulins)	5	106,157,590	64	666	270	396	59
Diabetes (SGLT2 inhibitors)	3	22,181,045	74	574	225	349	61
Diabetes (Short-acting insulins)	2	117,439,195	45	485	303	182	38
Erectile dysfunction (other)	1	4,742,000	54	117	50	66	56
Erythropoietin-stimulating agents	3	2,706,854	224	1987	1128	859	43
Epinephrine	2	6,016,942	66	352	293	59	17
Erectile dysfunction (PDE5 inhibitors)	2	31,848,176	84	357	234	123	34
Exocrine pancreatic insufficiency	2	6,346,323	107	1345	1060	284	21
Female hormone therapy	2	80,296,949	50	194	152	42	22
Fibrinolytics	2	2,640,336	147	631	501	130	21
Glaucoma treatments	5	49,709,883	49	182	103	79	43
Gonadotropins	3	454,213	420	1639	1479	160	10
Gout treatments	2	11,106,979	50	270	196	73	27
Hepatitis C treatments	3	792,021	993	27668	16101	11567	42
HIV treatments	19	17,522,590	97	1600	1161	439	27
Hypothyroid treatments	2	254,814,256	26	40	23	17	43
Inflammatory bowel disease treatments	8	21,386,038	83	733	567	167	23
Irritable bowel syndrome treatments	3	9,901,896	50	790	546	245	31
Immunosuppressants	5	4,031,515	92	703	588	115	16
Insomnia treatments	2	33,924,718	51	215	151	64	30

© 2021 Yeung K et al. *JAMA Network Open.*

Multiple sclerosis treatments	5	6,040,633	414	2287	1628	659	29
Migraine (Non-triptans)	1	236,275	64	371	193	178	48
Migraine (Triptans)	7	27,195,697	59	269	204	64	24
Nasal glucocorticoids	6	70,548,192	42	140	82	58	41
Overactive bladder treatments	3	28,444,394	47	236	181	55	23
Obesity	2	723,096	45	656	487	170	26
Oncology (Hormonal / GnRH)	1	377,669	79	2610	1017	1593	61
Oncology (Kinase inhibitors)	1	8,129	1	7260	7017	242	3
Oncology (Nucleoside metabolic inhibitors)	1	970,053	234	2127	1544	583	27
Ophthalmic treatments	3	15,430,998	68	800	480	321	40
Opioid dependence	1	1,315,142	20	235	135	101	43
Oral anticoagulants	2	55,014,011	69	540	315	225	42
Osteoporosis	8	88,298,275	65	250	182	68	27
Overactive bladder	3	36,546,264	61	354	190	164	46
Pain (Opioids)	2	1,814,395	29	332	192	140	42
Pain, Inflammation (Non-opioids)	8	155,816,940	54	333	238	95	29
Parkinson's disease treatments	3	6,788,464	71	404	373	32	8
Platelet aggregation inhibitors	3	116,757,720	53	255	230	26	10
Pulmonary arterial hypertension	1	147,367	56	2483	2428	55	2
Smoking cessation	1	10,695,148	73	198	162	36	18
Testosterone	5	16,100,863	66	478	348	130	27
Vaccines	4	876,175	45	135	114	21	16
Other	147	415,035,958	49	231	168	63	27

ADHD: attention deficit hyperactivity disorder, ARB: angiotensin II receptor blocker, Anti-TNF: anti-tumor necrosis factor, COPD: chronic obstructive pulmonary disease, DMARDs: disease-modifying antirheumatic drugs, DPP-4: dipeptidyl peptidase-4, GLP-1: glucagon-like peptide-1, GnRH: gonadotropin-releasing hormone, HIV: human immunodeficiency virus, PCSK9: proprotein convertase subtilisin/kexin type 9, PDE5: phosphodiesterase type 5; PPIs, proton pump inhibitors; SGLT2, sodium-glucose co-transporter-2

© 2021 Yeung K et al. *JAMA Network Open.*

eTable 5. Regression Coefficients From Primary Analysis, Overall Model, 2007-2013 and 2014-2018

**Regression parameter**	**Beta Coefficient (95% CI)**
Rebate	0.08 (0.03 to 0.13)
Commercial	0.56 (-2.68 to 3.81)
Uninsured	20.66 (0.93 to 40.39)
Commercial*Rebate	-0.08 (-0.13 to -0.03)
Uninsured*Rebate	0.56 (0.11 to 1.01)
Post_2014	-2.77 (-17.99 to 12.46)
Post_2014*Rebate	-0.02 (-0.09 to 0.05)
Commercial*Post_2014	8.62 (-12.06 to 29.29)
Uninsured*Post_2014	24.36 (-14.35 to 63.07)
Commercial*Post_2014*Rebate	0.02 (-0.07 to 0.12)
Uninsured*Post_2014*Rebate	-0.43 (-0.9 to 0.03)
Abilify	48.21 (12.7 to 83.72)
Acanya	-7.08 (-20.04 to 5.88)
Accolate	-21.47 (-32.88 to -10.05)
Accuneb	-23.03 (-46.03 to -0.04)
Aciphex	1.71 (-6.87 to 10.29)
Actonel	0.4 (-5.8 to 6.6)
Actos / Actoplus Met	6 (-0.16 to 12.15)
Acular / LS / PF	-33.81 (-65.04 to -2.58)
Aczone	-22.6 (-36.61 to -8.6)
Adderall XR	-27.74 (-38.6 to -16.89)
Admelog	-62.14 (-88.96 to -35.31)
Advair	-2.01 (-6.41 to 2.38)
Akne-Mycin	-7.7 (-33.01 to 17.62)
Aldara	-48.44 (-51.42 to -45.45)
AlleRx	-13.94 (-16.91 to -10.96)
AlleRx DF	-35.38 (-38.04 to -32.71)
Allegra / -D	-17.59 (-22.09 to -13.08)
Alora	-8.41 (-17.26 to 0.44)
Alphagan P	-11.92 (-24.32 to 0.49)
Alrex	33.36 (-27.23 to 93.96)
Altabax	-1.49 (-11.26 to 8.28)
Altace	-13.78 (-19.93 to -7.63)
Alvesco	-21.58 (-31.94 to -11.21)
Amaryl	-32.81 (-41.42 to -24.21)
Ambien / CR	-2.75 (-8.59 to 3.1)
Amitiza	-22.87 (-40.62 to -5.11)
Ampyra	-57.73 (-65.75 to -49.71)
Amrix	-18.16 (-36.83 to 0.51)
Androderm	30.68 (0.04 to 61.33)
Androgel	17.57 (-5.22 to 40.36)
Android	-3.28 (-6.18 to -0.38)

© 2021 Yeung K et al. *JAMA Network Open.*

Anoro Ellipta	-10.44 (-29.39 to 8.51)
Anusol-HC	-43.74 (-46.64 to -40.85)
Apidra	37.29 (15.62 to 58.97)
Aplenzin	12.46 (-18.21 to 43.13)
Apriso	-9.17 (-19.29 to 0.96)
Aranesp	543.61 (401.02 to 686.21)
Aricept	38.82 (14.93 to 62.7)
Arimidex	21.14 (-2.33 to 44.62)
Arixtra	29.34 (-11.95 to 70.63)
Armour Thyroid	-27.79 (-35.33 to -20.26)
Arnuity Ellipta	29.15 (-27.53 to 85.82)
Aromasin	-50.41 (-53.98 to -46.84)
Arthrotec	-7.07 (-29.12 to 14.97)
Asacol	15.1 (-7.13 to 37.33)
Asacol HD	46.75 (22 to 71.51)
Asmanex	-29.13 (-43.99 to -14.28)
Atacand / HCT	7.59 (-3.32 to 18.5)
Atelvia	11.92 (-14.95 to 38.8)
Ativan	-27.36 (-38.9 to -15.82)
Atralin	-9.82 (-44.69 to 25.05)
Atripla	42.47 (-70.15 to 155.1)
Augmentin / XR / ES-600	-18.41 (-22.49 to -14.33)
Auvi-Q	-35.08 (-61.2 to -8.96)
Avandamet	2.64 (-14.66 to 19.94)
Avandaryl	10.29 (-6.59 to 27.16)
Avandia	10.41 (-1.77 to 22.59)
Avapro / Avalide	0.12 (-5.48 to 5.72)
Avelox	6.06 (-1.91 to 14.03)
Avinza	9.16 (-21.63 to 39.94)
Avodart	4.88 (-2.12 to 11.87)
Avonex	326.41 (0.72 to 652.09)
Axiron	-33.74 (-92.38 to 24.89)
Azasan	-6.08 (-8.7 to -3.45)
Azilect	11.53 (4.47 to 18.59)
Azor	7.13 (-7.37 to 21.62)
Bactroban	-23.77 (-33.17 to -14.37)
Balacet 325	50.51 (47.08 to 53.94)
Baraclude	105.45 (-158.21 to 369.1)
Basaglar	-50.5 (-66.5 to -34.49)
Benicar / HCT	-0.29 (-4.54 to 3.96)
Bentyl	-39.11 (-59.06 to -19.16)
Benzaclin	-33.11 (-61.58 to -4.65)
Bepreve	-20.72 (-38.19 to -3.25)
Besivance	5.62 (-8.12 to 19.36)
Betaseron	137.78 (-128.63 to 404.2)

© 2021 Yeung K et al. *JAMA Network Open.*

Biaxin / XL	-26.46 (-38.52 to -14.39)
Bicillin	-36.47 (-44.21 to -28.73)
Boniva	-0.16 (-9.07 to 8.74)
Boostrix	-48.43 (-52.76 to -44.11)
Breo Ellipta	25.55 (-20.79 to 71.89)
Brevicon	-23.67 (-34.04 to -13.29)
Brilinta	23.55 (-37.83 to 84.93)
Bydureon	21.3 (-35 to 77.6)
Byetta	29.55 (5.52 to 53.58)
Bystolic	-2.85 (-8.77 to 3.07)
Caduet	16.62 (1.84 to 31.4)
Cambia	-2.91 (-28.3 to 22.48)
Campral	33.31 (-20.42 to 87.04)
Canasa	10.43 (-30.16 to 51.03)
Capital w/ Codeine	0.61 (-18.34 to 19.55)
Carac	11.11 (-14.49 to 36.7)
Carafate	-1.44 (-15.73 to 12.85)
Cardizem	-36.95 (-49.04 to -24.86)
Cardizem CD	-8.51 (-37.14 to 20.12)
Cardizem LA	14.53 (-12.38 to 41.44)
Casodex	77.1 (-42.93 to 197.12)
Celebrex	1.93 (-3.1 to 6.96)
Celexa	-5.63 (-54.98 to 43.72)
Cellcept	22.36 (-66.9 to 111.62)
Chantix	22.94 (11.13 to 34.75)
Cialis	34.89 (17.28 to 52.5)
Cimzia	1718.88 (1555.67 to 1882.08)
Clarinex / D	-13.17 (-17.82 to -8.51)
Cleocin	-18.13 (-62.09 to 25.84)
Cloderm	-12.06 (-27.94 to 3.81)
Colazal	9.1 (2.45 to 15.75)
Colcrys	-13.77 (-25.83 to -1.71)
Combigan	-4.14 (-19.71 to 11.42)
Combivir	-13.73 (-53.65 to 26.2)
Complera	-292.95 (-540.62 to -45.27)
Concerta	-0.36 (-9.09 to 8.38)
Condylox	-18.51 (-29.31 to -7.7)
Copaxone	344.26 (148.13 to 540.4)
Cordran Tape	-27.48 (-39.35 to -15.61)
Coreg / CR	-13.42 (-19.84 to -7)
Cosopt / Trusopt	-7.48 (-14.9 to -0.06)
Cotempla XR-ODT	-42.08 (-49.98 to -34.18)
Cozaar / Hyzaar	-0.53 (-8 to 6.95)
Creon	5.26 (-40.15 to 50.67)
Crestor	0.11 (-4.02 to 4.23)

© 2021 Yeung K et al. *JAMA Network Open.*

Crinone	7.62 (-39.29 to 54.52)
Cymbalta	3.02 (-3.07 to 9.12)
Cytomel	-7.86 (-25.23 to 9.51)
Daliresp	34.38 (-73.18 to 141.94)
Delatestryl	-4.39 (-19.44 to 10.66)
Delzicol	15.33 (-26.83 to 57.5)
Depakote	-17.15 (-26.85 to -7.46)
Descovy	40.06 (-114.08 to 194.19)
Detrol / LA	-5.01 (-11.01 to 0.98)
Dexilant	-7.33 (-16 to 1.34)
Dexpak	9.31 (-21.92 to 40.54)
Diastat / Acudial	158.69 (3.72 to 313.66)
Diflucan	-42.45 (-46.48 to -38.43)
Diovan / HCT	-3.72 (-6.96 to -0.48)
Diuril	-45.61 (-48.82 to -42.4)
Doryx	-16.34 (-32.06 to -0.62)
Dovonex	-16.43 (-29.4 to -3.45)
Duac / CS	-13.8 (-30.64 to 3.05)
Duexis	370.12 (-218.74 to 958.98)
Dulera	0.12 (-9.13 to 9.37)
Edecrin	-195.26 (-305.84 to -84.67)
Effexor / XR	-4.88 (-12.12 to 2.37)
Effient	9.11 (-6.81 to 25.02)
Efudex	36.3 (-25.01 to 97.61)
Elidel	-10.45 (-51.21 to 30.32)
Eliquis	19.3 (2.53 to 36.06)
Embeda	-54.89 (-58.15 to -51.62)
Emend	37.54 (-27.53 to 102.6)
Emtriva	4.38 (-0.48 to 9.25)
Enablex	-0.8 (-11.74 to 10.14)
Enbrel	141.76 (42.61 to 240.92)
Engerix-B	-9.22 (-12.7 to -5.73)
Entresto	48.36 (-68.21 to 164.93)
EpiPen	15.63 (0.74 to 30.51)
Epivir	92.79 (-46.09 to 231.67)
Epivir HBV	49.25 (12.71 to 85.79)
Epogen	-6.55 (-63.63 to 50.53)
Epzicom	-116.01 (-274.1 to 42.09)
Ertaczo	124.08 (86.79 to 161.36)
Estrace cream	11.56 (0.61 to 22.5)
Estrostep Fe / Tilia	-13.17 (-24.46 to -1.87)
Evista	19.07 (10.21 to 27.94)
Exalgo	-61.55 (-72.4 to -50.71)
Exelon	80.81 (0.42 to 161.2)
Exforge / HCT	-3.81 (-11.79 to 4.17)

© 2021 Yeung K et al. *JAMA Network Open.*

FML / Forte / S / S.O.P.	-51.5 (-62.28 to -40.72)
Factive	64.98 (-45.11 to 175.07)
Famvir	-14.03 (-26.43 to -1.63)
Farxiga / Xigduo	-15.17 (-41.33 to 10.98)
Fazaclo	-23.43 (-66.62 to 19.76)
Femara	20.39 (-2.24 to 43.01)
Femcon Fe	-1.17 (-17.87 to 15.54)
Femhrt	10.11 (-4.06 to 24.28)
Femring	52.02 (6.97 to 97.07)
Femtrace	1.88 (-1.52 to 5.29)
Fenoglide	-38.43 (-43.25 to -33.61)
Fentora	615.69 (458.92 to 772.45)
Fiasp	-32.18 (-55.46 to -8.9)
Flector	29.12 (8.92 to 49.32)
Flovent	-0.9 (-8.16 to 6.37)
Fluarix / FluLaval	-43.19 (-54.95 to -31.42)
Fluzone	-45.05 (-54.88 to -35.22)
Follistim AQ	176.11 (-63.77 to 415.99)
Foradil	-50.21 (-75.21 to -25.22)
Forteo	345.12 (187.29 to 502.95)
Fortesta	-28.57 (-36.67 to -20.46)
Fosamax / Plus D	-4.54 (-10.64 to 1.55)
Fragmin	230.65 (53.68 to 407.61)
Frova	0.96 (-28.13 to 30.04)
Fuzeon	-47.4 (-50.82 to -43.98)
Gabitril	-49.93 (-52.78 to -47.08)
Gelnique	-5.27 (-17.93 to 7.39)
Generess Fe	-29.08 (-41.82 to -16.33)
Geodon	-11.49 (-25.93 to 2.95)
Glucagon	-20.61 (-26.71 to -14.51)
Glumetza	-11.91 (-45.05 to 21.22)
Gonal-F	552.37 (-53.37 to 1158.1)
Gralise	-11.58 (-25.85 to 2.7)
Harvoni	157.99 (-1192.91 to 1508.88)
Hepsera	24.21 (21.62 to 26.8)
Humalog / Mix	-6.43 (-15.59 to 2.73)
Humira	234.8 (145.94 to 323.66)
Humulin / Mix	-18.06 (-23.15 to -12.97)
Hycodan	-32.44 (-37.9 to -26.98)
Hylatopic / Plus	-22.19 (-29.14 to -15.24)
Hyomax	-14.45 (-41.37 to 12.47)
Imitrex	35.12 (4.99 to 65.25)
Implanon / Nexplanon	-45.18 (-48.19 to -42.17)
Incivek	9512.2 (9290.98 to 9733.42)
Incruse Ellipta	-30.93 (-54.99 to -6.87)

© 2021 Yeung K et al. *JAMA Network Open.*

Infed	-128.63 (-162.64 to -94.62)
Intal	-45.76 (-52.27 to -39.25)
Intelence	-33.46 (-44.7 to -22.21)
Intuniv	-20.18 (-35.53 to -4.82)
Invega	12.68 (-38.96 to 64.31)
Invega Sustenna / Trinza	-32.79 (-39 to -26.57)
Invokana / Invokamet	35.21 (1.03 to 69.4)
Isentress	80.85 (-22.23 to 183.93)
Istalol	34.95 (-19.21 to 89.12)
Janumet / XR	3.47 (-9.59 to 16.52)
Januvia	-4.99 (-11.54 to 1.57)
Jardiance	-12.52 (-36.1 to 11.07)
Jentadueto	24.7 (19.06 to 30.33)
Kadian	-34.78 (-57.26 to -12.29)
Kaletra	-6.08 (-22.45 to 10.29)
Keppra / XR	-0.27 (-25.69 to 25.14)
Kombiglyze	-0.45 (-64.78 to 63.88)
Kytril	138.8 (103.74 to 173.87)
Lamictal / XR	16.44 (-4.29 to 37.16)
Lamisil	-20.99 (-54.86 to 12.88)
Lantus	4.71 (-2.43 to 11.85)
Latuda	6.03 (-38.9 to 50.97)
Lescol / XL	-8.34 (-16.83 to 0.14)
Levaquin / Floxin	-4.28 (-8.74 to 0.19)
Levemir	-17.11 (-25.8 to -8.41)
Levitra	1.29 (-10.06 to 12.65)
Levoxyl	-36.72 (-39.61 to -33.84)
Lexapro	1.29 (-2.69 to 5.27)
Lexiva	-59.44 (-66.5 to -52.39)
Lialda	33.9 (-12.25 to 80.05)
Librax	-30.35 (-45.98 to -14.72)
Lidoderm	0.26 (-12.09 to 12.61)
Linzess	-19.35 (-35.2 to -3.51)
Lo Loestrin Fe	-39.12 (-47.59 to -30.66)
Locoid / Lipocream	-5.33 (-29.66 to 19)
Lodosyn	17.83 (0.16 to 35.49)
Loestrin 24 Fe	-8.61 (-13.84 to -3.38)
Lotemax	-11.92 (-20.29 to -3.54)
Lovaza	-9.15 (-14.29 to -4)
Lovenox	82.44 (27.17 to 137.71)
Lumigan	4.88 (-9.15 to 18.91)
Lunesta	1.32 (-14.45 to 17.08)
Lupron	-29.77 (-125.3 to 65.75)
Luvox CR	14.57 (8.23 to 20.91)
Lyrica	-0.67 (-7.87 to 6.52)

© 2021 Yeung K et al. *JAMA Network Open.*

Maxalt	-0.59 (-11.56 to 10.37)
Mestinon	-20.55 (-23.21 to -17.88)
Minastrin 24 Fe	-39.86 (-49.42 to -30.3)
Moban	-41.83 (-45.29 to -38.37)
Moviprep	-9.5 (-16.4 to -2.6)
Multaq	68.23 (19.82 to 116.63)
Mydayis	-42.64 (-54.94 to -30.34)
Myfortic	43.59 (2.94 to 84.25)
Myrbetriq	16.04 (-1.69 to 33.76)
Namenda	3.85 (-7.85 to 15.54)
Namenda XR	57.42 (29.28 to 85.56)
Nasacort	-1.48 (-9.71 to 6.76)
Nascobal	-29.54 (-66.59 to 7.51)
Nasonex	-11.71 (-15.8 to -7.62)
Nephro-Vite Rx	-15.42 (-28.01 to -2.84)
Nephrocaps	-36.92 (-46.27 to -27.56)
Nexium	-12.28 (-19.25 to -5.31)
Niaspan	-11.61 (-17.16 to -6.06)
Norvir	-21.95 (-58.54 to 14.65)
Novolin	-24.93 (-33.59 to -16.28)
Novolog / Mix	0.58 (-13.82 to 14.97)
Noxafil	-110.1 (-152.66 to -67.55)
Nucynta / ER	-22.96 (-60.78 to 14.87)
Nuvaring	-22.94 (-28.63 to -17.26)
Nuvigil	4.24 (-17.24 to 25.71)
Omnaris	3.23 (-9.09 to 15.55)
Onexton	-24.84 (-38.03 to -11.64)
Onglyza	0.79 (-23.97 to 25.55)
Opana	-43.3 (-46.89 to -39.72)
Opana ER	-19.84 (-53.56 to 13.87)
Osmoprep	2.75 (-16.89 to 22.39)
Ovidrel	27.2 (3.79 to 50.61)
Oxsoralen / -Ultra	168.54 (149.17 to 187.92)
Oxtellar XR	61.48 (36.11 to 86.85)
Oxytrol	-20.93 (-36.41 to -5.44)
Ozempic	-25.9 (-70.57 to 18.78)
Pataday / Patanol / Pazeo	-7.14 (-20.1 to 5.82)
Paxil / CR	32.81 (-24.29 to 89.91)
Pennsaid 2%	10.82 (-114.68 to 136.32)
Pentasa	60.36 (-18.69 to 139.42)
Pepcid suspension	-41.51 (-45.33 to -37.69)
Plavix	2.96 (-2.21 to 8.12)
Plendil / Lexxel	-46.06 (-61.35 to -30.77)
Pred Forte / G / Mild	8.32 (-12.27 to 28.91)
Premarin family	-1.35 (-5.78 to 3.08)

© 2021 Yeung K et al. *JAMA Network Open.*

Preque 10	-1.31 (-7.96 to 5.35)
Prevacid	-2.51 (-11.23 to 6.21)
Prezista / Prezcobix	-12.17 (-48.82 to 24.48)
Pristiq	-1.14 (-9.66 to 7.38)
ProAir	-24.47 (-28.38 to -20.56)
Procrit	-51.77 (-91.87 to -11.67)
Prograf	10.09 (-27.38 to 47.57)
Prolensa	14.08 (-10.92 to 39.08)
Prolia	64.27 (-15.56 to 144.11)
Propecia	119.77 (94.7 to 144.84)
Protonix	-7.32 (-21 to 6.36)
Protopic	4.5 (-17.08 to 26.08)
Proventil	-19.83 (-23.72 to -15.94)
Provigil	13.6 (-4.11 to 31.31)
Pulmicort	5.56 (-12.83 to 23.94)
Pylera	25.82 (-16.14 to 67.77)
Qvar	-9.2 (-20.03 to 1.62)
Ranexa	-3.96 (-19.71 to 11.8)
Rapaflo	9.33 (-7.97 to 26.63)
Rapamune	41.48 (21.81 to 61.15)
Razadyne / ER / Reminyl	-5.71 (-40.4 to 28.99)
Rebif	728.51 (301.48 to 1155.53)
Reclast	-26.04 (-49.96 to -2.11)
Rectiv	-19.87 (-35.54 to -4.2)
Relenza	1.14 (-10.81 to 13.09)
Relpax	2.78 (-11.23 to 16.79)
Remicade	528.76 (518.18 to 539.35)
Renagel / Renvela	19.81 (-33.37 to 72.98)
Requip / XL	-11.36 (-18.82 to -3.91)
Restasis	12.36 (-14.96 to 39.67)
Retin-A Micro	15.98 (-30.96 to 62.92)
Revatio	3.27 (-66.09 to 72.63)
Reyataz / Evotaz	-15.44 (-117 to 86.12)
Rhinocort	-9.53 (-15.46 to -3.6)
Risperdal	17.43 (-15.5 to 50.35)
Ritalin / Focalin	-1.74 (-13.13 to 9.64)
Saphris	-20.71 (-47.66 to 6.24)
Sarafem	-13.79 (-19.51 to -8.07)
Savella	1.76 (-33.52 to 37.03)
Saxenda	-23.34 (-48.62 to 1.93)
Semprex-D	-30.51 (-37.67 to -23.34)
Serevent	-2.85 (-18.58 to 12.89)
Seroquel IR	-4.48 (-14.35 to 5.4)
Seroquel XR	46.49 (-18.19 to 111.17)
Shingrix	-23.19 (-47.14 to 0.76)

© 2021 Yeung K et al. *JAMA Network Open.*

Singulair	-1.49 (-5.65 to 2.67)
Skelaxin	3.77 (-8.64 to 16.18)
Solodyn	63.43 (-13.37 to 140.22)
Sonata	-26.8 (-51.3 to -2.29)
Soriatane	26.43 (-1.78 to 54.65)
Spectracef	-38.99 (-44.68 to -33.31)
Sprycel	-69.85 (-77.94 to -61.75)
Stalevo / Comtan	61.03 (21.52 to 100.54)
Strattera	15.37 (-5.23 to 35.96)
Stribild	-52.49 (-58.91 to -46.06)
Suboxone Film	-33.83 (-47.63 to -20.03)
Sumavel Dosepro	25.46 (-11.18 to 62.09)
Sustiva	10.88 (-18.4 to 40.15)
Symbicort	-2.21 (-9.07 to 4.65)
Symbyax	-36.71 (-50.63 to -22.8)
Symlin	30.54 (-9.08 to 70.16)
Synalar	-48.91 (-51.56 to -46.27)
Synthroid	-23.55 (-26.92 to -20.18)
Taclonex	9.3 (-9.11 to 27.71)
Tamiflu	3.25 (-1.71 to 8.21)
Taytulla	-34.66 (-44.3 to -25.02)
Tegretol / XR	16.3 (-13.6 to 46.19)
Tekturna / HCT / Tekamlo / Valturna	-5.27 (-14.17 to 3.63)
Testim	-4.6 (-17.45 to 8.25)
Theo-24	113.13 (106.44 to 119.81)
Timoptic / XE	-13.28 (-48.43 to 21.87)
Topamax	7.4 (-7.23 to 22.03)
Toprol-XL	-21.33 (-24.51 to -18.14)
Toujeo	-27.23 (-45.67 to -8.79)
Toviaz	60.36 (0.79 to 119.94)
Tradjenta	-16.27 (-28.09 to -4.45)
Travatan / Z	-0.25 (-11.13 to 10.64)
Tresiba	-32.32 (-51.83 to -12.82)
Tretin-X	70.98 (-24.45 to 166.41)
Treximet	17.22 (-7.24 to 41.68)
Tribenzor	-23.44 (-30.71 to -16.16)
Tricor / Trilipix	-4.6 (-9.89 to 0.69)
Trileptal	2.89 (-42.36 to 48.14)
Trintellix	0.65 (-22.34 to 23.64)
Trokendi XR	160.04 (-49.95 to 370.02)
Trulicity	0.3 (-41.73 to 42.33)
Truvada	18.06 (-29.63 to 65.75)
Tudorza	-10.81 (-31.08 to 9.45)
Uceris	454.08 (120.24 to 787.93)

© 2021 Yeung K et al. *JAMA Network Open.*

Uloric	9.35 (-4.27 to 22.97)
Ultram ER	24.28 (-37.14 to 85.7)
Uroxatral	7.8 (-4.45 to 20.06)
Valcyte	43.29 (-94.55 to 181.12)
Valtrex	-1.23 (-17.53 to 15.07)
Vanos	27.84 (-1.68 to 57.35)
Vascepa	-19.22 (-30.86 to -7.58)
Ventolin	-25.17 (-29.54 to -20.79)
Veramyst	-16.75 (-21.96 to -11.54)
Vesicare	-3.01 (-10.44 to 4.43)
Vfend	-17.41 (-21.73 to -13.09)
Viagra	24.75 (12.49 to 37.01)
Victoza	13.94 (0.48 to 27.41)
Victrelis	-21.25 (-84.72 to 42.21)
Viibryd	19.1 (0.32 to 37.89)
Vimovo	-38.96 (-67.56 to -10.36)
Vimpat	51.68 (-26.36 to 129.72)
Viread	-6.36 (-37.97 to 25.26)
Voltaren gel	-18.66 (-24.46 to -12.86)
Vytorin	2.74 (-1.76 to 7.25)
Vyvanse	18.36 (4.45 to 32.27)
Welchol	0.74 (-12.06 to 13.53)
Xalatan	-2.71 (-9.09 to 3.67)
Xarelto	1.72 (-12.36 to 15.8)
Xeloda	166.25 (-152.85 to 485.35)
Xenical	2.19 (-9.97 to 14.34)
Xifaxan	34.91 (-17.26 to 87.08)
Xiidra	33.99 (-14.8 to 82.79)
Xolair	91.1 (-30.25 to 212.45)
Xopenex HFA	-21.2 (-29.03 to -13.38)
Xopenex solution	43.14 (-37.32 to 123.6)
Xtampza ER	-28.48 (-44.77 to -12.19)
Yaz / Yasmin / Safyral / Beyaz	-11.18 (-14.8 to -7.55)
Zanaflex	-2.41 (-24.06 to 19.23)
Zegerid	4 (-3.21 to 11.22)
Zelapar	-36.45 (-39.67 to -33.24)
Zemplar	-29.52 (-44.37 to -14.67)
Zenpep	232.59 (-58.38 to 523.57)
Zetia	1.39 (-3.17 to 5.95)
Zetonna	84.37 (5.7 to 163.03)
Ziagen	19.95 (-15.3 to 55.2)
Ziana	-16.17 (-44.06 to 11.71)
Zipsor	-28.83 (-50.84 to -6.82)
Zirgan	-8.62 (-15.86 to -1.38)
Zoladex	276.74 (181.89 to 371.6)

© 2021 Yeung K et al. *JAMA Network Open.*

Zomig	1.19 (-24.19 to 26.58)
Zostavax	9.94 (-14 to 33.87)
Zovirax topical	-12.81 (-22.46 to -3.17)
Zyclara	59.96 (-85.9 to 205.83)
Zyflo / CR	110.16 (95.95 to 124.37)
Zylet	22.62 (-12.3 to 57.54)
Zyprexa	26.41 (-4.45 to 57.27)
Zyrtec	-5.17 (-10.57 to 0.23)
2008	-1.44 (-4.3 to 1.42)
2009	-3.48 (-6.1 to -0.85)
2010	0.23 (-2.85 to 3.31)
2011	-0.09 (-3.48 to 3.3)
2012	4.13 (-2.17 to 10.43)
2013	-2.01 (-7.8 to 3.78)
2014	3.24 (-5.26 to 11.75)
2015	-4.35 (-12.09 to 3.38)
2016	-2.08 (-11.88 to 7.72)
2017	-1.79 (-9.65 to 6.07)
constant	47.64 (44.04 to 51.24)

* signifies coefficient for interaction term (e.g. Commercial*Rebate = Commercial times Rebate)
CI, confidence interval

eTable 6. Total Out-of-Pocket Costs for Single-Source Branded Prescriptions Per Branded Drug User Per Year[a]

**2007-2013**	**Out-of-pocket costs per user per year (95% CI), 2018 US dollars**		
	With Observed Rebates[b]	With Zero Rebates[c]	Difference
Overall	421 (411 to 431)	387 (378 to 395)	34 (32 to 36)
By coverage type			
Medicare	527 (508 to 546)	457 (441 to 473)	70 (66 to 74)
Commercial	335 (327 to 344)	337 (328 to 345)	-1 (-1 to -1)
Uninsured	672 (609 to 735)	462 (415 to 510)	210 (185 to 234)
**2014-2018**	**Out-of-pocket costs per user per year (95% CI), 2018 US dollars**		
	With Observed Rebates[b]	With Zero Rebates[c]	Difference
Overall	369 (357 to 380)	302 (293 to 312)	66 (62 to 70)
By coverage type			
Medicare	414 (398 to 431)	329 (316 to 342)	85 (79 to 91)
Commercial	314 (299 to 329)	280 (266 to 294)	34 (32 to 37)
Uninsured	682 (578 to 786)	471 (388 to 555)	211 (178 to 243)

[a] Total out-of-pocket costs for single-source branded prescriptions per user per year appear to have decreased for Medicare and commercial insurance patients as the number of branded prescriptions filled per year have also decreased (see eTable 2).

[b] i.e., factual estimates: the model-adjusted estimate of out-of-pocket costs with observed rebates

[c] i.e., counterfactual estimates: the model-adjusted estimate of out-of-pocket costs if rebates were set to zero

CI, confidence interval

© 2021 Yeung K et al. *JAMA Network Open.*

eTable 7. Estimated Change in Dependent Variable Given $1 Increase in Independent Variable, 2007-2013 and 2014-2018

**2007-2013** **Dependent variable**	**Independent variable**	**Additional Covariate**[a]	**Change in dependent variable given $1 increase in the independent variable (95% CI), 2018 US dollars**[a]
List price	Rebates	-	2.75 (2.55 to 2.96)
Out-of-pocket cost	List price	-	0.02 (0.01 to 0.03)
Out-of-pocket cost[b]	Rebates	-	0.07 (0.02 to 0.12)
Out-of-pocket cost	Rebates	List price	0.00 (-0.07 to 0.07)
**2014-2018** **Dependent variable**	**Independent variable**	**Additional Covariate**[a]	**Change in dependent variable given $1 increase in the independent variable (95% CI), 2018 US dollars**[a]
List price	Rebates	-	2.09 (1.96 to 2.22)
Out-of-pocket cost	List price	-	0.02 (0.01 to 0.03)
Out-of-pocket cost[b]	Rebates	-	0.04 (0.02 to 0.06)
Out-of-pocket cost	Rebates	List price	-0.01 (-0.04 to 0.02)

[a] As with main analyses, these analyses included drug and year indicators and interactions for coverage type and pre and post 2014
[b] Identical model specification as primary analysis (i.e., Table 2 results), expressed here as estimated change in out-of-pocket cost given $1 increase in estimated rebates
CI, confidence interval

© 2021 Yeung K et al. *JAMA Network Open.*

eTable 8. Sensitivity Analyses of Estimated Out-Of-Pocket Costs With 2009-2013 Preperiod and Alternative Rebate Definitions

**eTable 8a** **2009-2013**	**Out-of-pocket costs per prescription (95% CI), 2018 US dollars**		
	**With Rebates**[a]	**Without Rebates**[b]	**Difference**
Using 2009-2013 instead of 2007-2013 as the pre-period	54 (53 to 54)	49 (49 to 50)	4 (4 to 5)
**eTable 8b** **2007-2013**	**Out-of-pocket costs per prescription (95% CI), 2018 US dollars**		
**Rebate Definitions**	**With Rebates**[a]	**Without Rebates**[b]	**Difference**
Rebate = list price minus the greater of net or FSS prices	52 (51 to 53)	49 (49 to 50)	3 (2 to 3)
Rebate = gross price minus net price	52 (51 to 52)	50 (49 to 51)	2 (2 to 2)
**eTable 8c** **2014-2018**	**Out-of-pocket costs per prescription (95% CI), 2018 US dollars**		
**Rebate Definitions**	**With Rebates**[a]	**Without Rebates**[b]	**Difference**
Rebate = list price minus the greater of net or FSS prices	61 (60 to 62)	53 (52 to 54)	8 (7 to 8)
Rebate = gross price minus net price	61 (60 to 62)	52 (51 to 53)	9 (8 to 9)

[a] Factual estimates: the model-adjusted estimate of out-of-pocket costs in the presence of the rebates

[b] Counterfactual estimates: the model-adjusted estimate of out-of-pocket costs in the absence of the rebates

FSS: drug prices from the Federal Supply Schedule reported by the United States Department of Veterans Affairs National Acquisition Center; CI, confidence interval

© 2021 Yeung K et al. *JAMA Network Open.*

eTable 9. Association of Rebates With Probability of Out-of-Pocket Costs and Increase in Out-of-Pocket Costs[a]

**2007-2013**	**Probability of incurring out-of-pocket costs, (95% CI)[b]**	**Increase in out-of-pocket costs, given non-zero out-of-pocket costs, % (95% CI)[c]**
Overall	0.00 (0.00 to 0.01)	0.32 (0.30 to 0.34)
By coverage type		
Medicare	0.01 (0.00 to 0.01)	0.39 (0.36 to 0.42)
Commercial	0.00 (0.00 to 0.01)	0.25 (0.23 to 0.28)
Uninsured	-0.01 (-0.03 to 0.01)	0.42 (0.31 to 0.53)
**2014-2018**	**Probability of incurring out-of-pocket costs, (95% CI)[b]**	**Increase in out-of-pocket costs, given non-zero out-of-pocket costs, % (95% CI)[c]**
Overall	0.00 (-0.01 to 0.01)	0.19 (0.16 to 0.22)
By coverage type		
Medicare	0.00 (-0.01 to 0.02)	0.23 (0.19 to 0.26)
Commercial	0.00 (-0.01 to 0.02)	0.15 (0.12 to 0.18)
Uninsured	0.01 (-0.04 to 0.05)	0.15 (0.00 to 0.31)

[a] Presented as elasticity estimates: percent change in out-of-pocket costs associated with 1% change in estimated rebates
[b] Probability of incurring out-of-pocket costs: i.e., extensive margin
[c] Increase in out-of-pocket costs, given non-zero out-of-pocket costs: i.e., intensive margin
CI, confidence interval

© 2021 Yeung K et al. *JAMA Network Open.*

eReferences

1. Hernandez I, San-Juan-Rodriguez A, Good CB, Gellad WF. Changes in list prices, net prices, and discounts for branded drugs in the US, 2007-2018. JAMA 2020;323:854-62.

© 2021 Yeung K et al. *JAMA Network Open.*
